# Therapeutic Notes

**Published:** 1903-01

**Authors:** Nelson W. Wilson

**Affiliations:** Buffalo, N.Y.


### PROGRESS IN MEDICAL SCIENCE.

### Therapeutic Notes.

EDITED BY NELSON W. WILSON, M.D., Buffalo, N.Y.

### THE PREPARATION OF KOUMYSS.

ONCE in awhile it is advisable to make koumyss, on which occasions the following method of preparation as given by H. V. Sachse, in How to Cook for the Sick, is the best:

Heat two quarts of milk to 100° F., or pasteurise and cool to 100° F. Dissolve one-third of a yeast-cake in two tablespoonfuls of the milk, boil two tablespoonfuls of sugar with three tablespoonfuls of cold water, mix all together and bottle at once. Cork firmly and stand in a warm (70° F.) place over night. Next morning place the bottles carefully on their sides in a cool place for twenty-four hours. Open with a tap, or use ordinary beer-bottles.

### FOR ACUTE RHEUMATISM.

F. L. SATTERLEE, in Rheumatism and Gout, goes into the treatment of acute rheumatism very thoroughly and lays down a routine plan which is, in many cases, really remarkable for the results it achieves. In two cases recently treated, according to Satterlee's ideas there was a most gratifying outcome. In the beginning he gives a chologogue for the reason that the bile in rheumatism is always acid when it should be alkaline in reaction. The acidity he ascribes to biliary stasis and uric acid accumulation. The chologogue given is as follows:

| | | | |
|---|---|---|---|
| ℞ | Euonymin | 0.015 | gr. ¼ |
| | Podophyllin | 0.008 | gr. ⅛ |
| | Aloin | 0.008 | gr. ⅛ |

Dose—One such tablet night and morning as required.

Now, alkalies are indicated and the best are the sodium salts. The best borne combination is:

| | | | |
|---|---|---|---|
| ℞ | Benzoate of lithia | 2.0 | ʒ ss. |
| | Bromide of soda | 8.0 | ʒ ii. |
| | Carbonate of potash | 8.0 | ʒ ii. |
| | Acetate of potash | 45.0 | ℥ iss. |
| | Phosphate of soda | 15.0 | ℥ ss. |
| | Syr. ginger, peppermint water, equal parts of each to make | 180.0 | ℥ vi. |

Mix. Dose—Two to four teaspoonfuls in water every four to six hours after food.

If deemed advisable bicarbonate of soda may be given, or this alkaline mixture:

| | | | |
|---|---|---|---|
| ℞ | Bicarbonate of potash | 12.0 | ʒ iii. |
| | Distilled water | 240.0 | ℥ viii. |

Mix. Dose—Teaspoonful to half an ounce of fresh lemon juice, to be drunk while effervescing.

It is important to remember to keep up the alkalies until the urine and sweat do not show acid reaction to blue litmus paper. When this stage has been reached small doses will serve to maintain the proper condition of alkalinity of the system. Salicylates are never employed, for the reason that they tend to produce relapses, cardiac depression and cerebral and gastric irritation. In the event of fever use antipyrin, antifebrin or phenacetin with great caution. To give quinine is worse than giving no treatment at all. It is absolutely and hopelessly useless. If the antipyretics are not sufficiently analgesic, and in the majority of cases they will be sufficient, morphine may be used, especially when natural and painless sleep is urgently needed by the patient. Then this may be given at one dose:

| | | |
|---|---|---|
| Sulphate of morphine | 0.015 | gr. ¼ |
| Bromide of potash | 2.0 | gr. xxx. |
| Chloral hydrate | 1.3 | gr. xx. |
| Syrup of orange peel | 8.0 | ʒ ii. |
| Water, to make | 30.0 | ℥ i. |

Vichy is the most serviceable of the mineral waters as a vehicle and Rubinat Llorach acts best on the liver. By this time the acute symptoms have subsided and now the heart needs tone. There are several mixtures suggested by various authors for this stage, but the best are these:

| | | | |
|---|---|---|---|
| ℞ | Aromatic spirits of ammonia | 90.0 | ℥ iii. |
| | Carbonate of ammonia | 4.0 | ʒ i. |
| | Tinct. cardamom | 30.0 | ℥ i. |
| | Tinct. nux vomica | 12.0 | ʒ iii. |

Mix. Dose—One teaspoonful in water three times a day.

| | | | |
|---|---|---|---|
| ℞ | Tinct. chloride of iron | 15.0 | ℥ ss. |
| | Tinct. nux vomica | 8.0 | ʒ ii. |
| | Dil. phosphoric acid | 8.0 | ʒ ii. |
| | Syrup orange peel | 30.0 | ℥ i. |
| | Elix. calisaya | 60.0 | ℥ ii. |

Mix. Dose—One teaspoonful in water half an hour before meals.

As a rule one will find excessive thirst. This is best met by the free use of lemonade or water. As to diet, it should be limited largely to milk, chicken or clam broths, and vegetable soups. When the return to solid food is permitted white meat only should be given at first with such vegetables as celery and asparagus.

The majority of patients will not only ask for a liniment but in many cases demand it and not feel satisfied unless they have a topical application. The best is this:

| | | | |
|---|---|---|---|
| ℞ | Tinct. aconite root | 15.0 | ℥ ss. |
| | Tinct. arnica | 15.0 | ℥ ss. |
| | Chloroform | 30.0 | ℥ i. |
| | Camphorated soap liniment | 60.0 | ℥ ii. |

Mix. Directions—Apply on lint or rub joint well and cover with cotton.

In closing, great stress is laid on the efficacy of blistering when the inflammation results in effusion into a joint.

---

### TUBERCULOSIS IN CHILDHOOD.

RACHFORD (*Archives of Pediatrics*) says the importance of diet becomes greater the younger the victim, and the necessity for artificially feeding infants complicates the problem. In cases under a year old he begins by feeding Nestle's food alone, and when the stools become normal adds cod-liver oil, pure or in emulsion, to several of the feedings each day. As the patient's condition improves, he gradually substitutes modified cow's milk. He also adds the white of one egg to one bottle of the food, and the yolk of an egg to another. In older children milk and cod-liver oil are the staples, and fresh eggs, good beef and poultry, cereals, fresh fruits, and well-cooked vegetables may be added as age permits. Fresh air and sunshine should be obtained for as much of the day as possible. He uses the following inunction:

| | | | |
|---|---|---|---|
| ℞ | Guaiacol | 4.00 | ʒ j. |
| | Lanolin | 8.00 | ʒ ij. |
| | Lard | 20.00 | ʒ v. |

M. Sig.: One level teaspoonful to be rubbed into chest daily at bedtime. This may be applied also over tuberculous lymph-nodes or over the abdomen in tuberculous peritonitis. The carbonate of guaiacol may be given internally, though creosote is preferred. Creosote may be given in capsule or by inhalation.

| | | | |
|---|---|---|---|
| ℞ | Tinct. gentian | 6.00 | ʒ iss. |
| | Creosote | 2.00 | ʒ ss. |

M. Sig.: Three to five drops in a capsule and take every six hours, followed by a drink of milk.

| | | | |
|---|---|---|---|
| ℞ | Tinct. camph. opium | 8.00 | ʒ ij. |
| | Creosote | 8.00 | ʒ ij. |
| | Alcohol | 16.00 | ʒ iv. |

M. Sig.— Five to fifteen drops in creosote inhaler; use fifteen minutes three times a day. The iodide of iron, arsenic and malt containing diastase, may each have their indications.

---

### BRONCHITIS WITH BRONCHOPLEGIA.

A. MARTINET (*La Presse Médicale*) says in treating bronchitis the two general indications are to lessen the cough and diminish the bronchial secretion. But a third indication, sometimes of great importance, is to combat bronchoplegia, the paralysis of the smooth muscles of the bronchi. This may be due to the toxins of certain infectious diseases, diphtheria, influenza, and the like, or may be produced by mechanical causes in the course of chronic bronchitis. There is gradual progressive dyspnea with ineffectual cough, accumulation of secretions and congestion. In such a condition an opiate or other sedative might do great harm, drugs that will stimulate the nervous system and reawaken the bronchial contractility being required. A cardio-tonic and hemostatic pill of value is:

| | | | |
|---|---|---|---|
| ℞ | Ext. hyoscyami | 0.3 | gr. v. |
| | Quin. sulph. | 1.5 | gr. xxiv. |
| | Ergotin | 3.0 | gr. xlv. |

M. Ft. pil. · No. xxx. Six or eight pills a day.

Rénaut's treatment of bronchitis is to give balsamics (terpin, syrup of tolu or Canada balsam, Venice turpentine, and the like) for the first four days; then for three days to give ergotin in suppository with opium or hyoscyamus, and at the same time pills of

| | | | |
|---|---|---|---|
| ℞ | Ext. hyoscyami | 0.3 | gr. v. |
| | Terpin | 3.0 | gr. xlv. |
| | Ergotin | 1.5 | gr. xxiv. |

M. Div. in pill No. xxx. Two or three a day.

In the bronchoplegia of grip, strychnin is the most useful remedy:

| | | | |
|---|---|---|---|
| ℞ | Strych. sulph. | 0.03 | gr. ss. |
| | Ergotin | 1.5 | gr. xxiv. |
| | Quinin. sulph. | 3.0 | gr. xlv. |

M. Div. in pil. No. xxx. Take every two hours with a hot infusion sweetened with syrup of tolu and containing a teaspoonful of old brandy.

INHALATIONS OF ANTISEPTICS IN TUBERCULOSIS.

Ever since the discovery of the specific cause of tuberculosis, constant efforts have been made to treat the disease by attacks on the bacilli, writes H. C. Wood, Jr., in *American Medicine.* The futility of giving antiseptics by the mouth with the expectation that they could reach the lungs through the blood, becoming evident with advancing knowledge, attempts have been made to reach the lungs directly through the trachea. The methods which have been employed to introduce drugs into the lungs are intratracheal injections, atomisation of watery or oily solutions, and converting the antiseptics into respirable gases. The last of these has received, recently, considerable attention, especially among German physicians.

One of the most recent contributions on this method of treating tuberculosis is a long and interesting paper by Rudolph, in which it is claimed that the failure of the treatment to achieve popularity is owing to deficiencies in apparatus. He points out the faults of the various appliances used for the purpose, including the steam atomiser of Siegle, the simple but useful device on the principle of the washbottle, and the complicated arrangement of Jahr, ending with a description of a new model which excels them all, at least in complexity. An examination of the instrument is better than a lengthy description of this apparatus, but we would point out that it is fundamentally a series of washbottles, connected with long, spiral tubes, the advantage of which is not apparent, nor is it mentioned in the text. The only real difference in Rudolph's apparatus from the common bottle-inhaler is that the air is forced through by a rubber bulb instead of being drawn through by inspiratory force, and as complex as it is, we cannot see why the same results could not be procured by simply attaching a double bulb to the bottle-inhaler.

Nevertheless the inventor has demonstrated that the air that comes out through his apparatus has distinct germicidal power if the bottles are filled with a volatile antiseptic, as guaiacol or acetic acid. He has also shown, chemically, that the vapors will pass into all portions of the lung of an animal breathing this air. He offers, however, no proof of the really essential question as to whether the vapors are not so diluted by the residual air of the lungs as to lose their germicidal properties. In fact, his own statements tend to show that they do lose their powers, for he affirms that his patients continued to expectorate tubercle bacilli even after a prolonged course of treatment. It is our belief that none of the present known disinfectants can be introduced into the lungs in sufficient quantity to be active without exercising a deleterious effect on the delicate pulmonary structure or causing a general intoxication; and we must further confess to grave doubts that any such antiseptic will ever be discovered, because all such bactericides are of necessity protoplasmic poisons and bound to affect the protoplasm of the patient as well as of the bacterium. Any improvement which follows the inhalation treatment of tuberculosis is more justly attributable to the effect of the drug on the mucous membranes than on the tubercle bacilli.

